# Survey of normalized CTDI_vol_ values across four major computed tomography vendors for use in the MIRDct software

**DOI:** 10.1002/acm2.70473

**Published:** 2026-01-26

**Authors:** Laura E. Dinwiddie, Jared M. Baggett, James M. Kofler, Cameron B. Kofler, Daniel J. Long, Robert J. Dawson, Stefan K. Wehmeier, Yitian Wang, Juan C. Ocampo‐Ramos, Lukas M. Carter, Harry Marquis, Gunjan Kayal, Adam L. Kesner, Wesley E. Bolch

**Affiliations:** ^1^ J. Crayton Pruitt Family Department of Biomedical Engineering University of Florida Gainesville Florida USA; ^2^ Medical Physics Graduate Program, College of Medicine University of Florida Gainesville Florida USA; ^3^ Department of Radiology Mayo Clinic Jacksonville Florida USA; ^4^ Department of Radiology University of Chicago Chicago Illinois USA; ^5^ Memorial Sloan Kettering Cancer Center New York New York USA; ^6^ MD Anderson Cancer Center Department of Imaging Physics Houston Texas USA

**Keywords:** computed tomography dose index, dose estimation, dosimetry

## Abstract

**Background:**

Computed tomography (CT) is an essential imaging modality for disease diagnosis, treatment efficacy, and image‐based guidance of various medical procedures. The locally deposited radiation dose in tissues, as estimated by the computed tomography dose index (CTDI), can vary considerably across exposures delivered by CT scanners from different vendors, even if the scans are performed using similar technique factors, such as tube potential and tube current. The volumetric CTDI (CTDI_vol_) is a common dose metric that reports an average radiation dose (in mGy) delivered to a specific volume within a test phantom. The CTDI_vol_ is important in dosimetry applications as the organ absorbed dose within the patient has been shown to scale in near‐linear proportion, creating a basis for comparing organ doses across different scan protocols and scanner models.

**Purpose:**

To develop a database of tube current‐time product (mAs) normalized CTDI_vol_ values for currently utilized CT scanner models for each of the four primary CT vendors for use in the MIRDct organ dosimetry software available at MIRDsoft.org. This data forms the basis of the MIRDct code, which reports organ doses across a range of computational phantoms based upon axial organ dose coefficient libraries generated through Monte Carlo radiation transport for a reference CT scanner. Organ doses delivered by alternate CT scanner vendors and models may then be reported using ratios of normalized CTDI_vol_ values under similar technique factors.

**Methods:**

Scanners were selected from four major CT manufacturers: Philips Healthcare, GE Healthcare, Canon Medical Systems, and Siemens Healthineers. Technique parameters were also selected for each scanner that closely matched values used in the generation of an equivalent CT source term (small to large bowtie filters; 80–140‐kVp tube voltage; and 10‐mm to 40‐mm beam collimation). For each scanner chosen, the appropriate technique factors and protocols were selected, and the console‐reported CTDI_vol_ values were recorded and normalized to a set value of 100 mAs. The normalized CTDI_vol_ data collected for use within the MIRDct code were analyzed for noticeable patterns, features, and trends, and were compared to similar normalized CTDI_vol_ datasets used within the National Cancer Institute NCICT software and the Virtual Phantoms, Inc. VirtualDose software.

**Results:**

For all given CT scanner and technique factor combinations, there was strong agreement in normalized CTDI_vol_ values across all three codes: between 0% and 12% difference for the compared scanners. Ratios of CTDI_vol_ values for various CT scanner vendors and models to the corresponding CTDI_vol_ values for the MIRDct reference scanner (Cannon Aquilion One Genesis) were also compared on the basis of either the 16‐cm head PMMA phantom or the 32‐cm body PMMA phantom. The mean quotient of these normalized CTDI_vol_ ratios (head ratios to body ratios) was found to be approximately 1.06, and thus either ratio may be applied in reporting patient organ dose by MIRDct.

**Conclusions:**

A database of normalized CTDI_vol_ (mGy/100 mAs) was created for 17 models of CT scanners from four manufacturers at varying tube potentials, collimations, x‐ray bowtie filters, and phantom sizes for use in the MIRDct software.

## 1 INTRODUCTION

Computed tomography (CT) is a foundational tool in modern medicine and is employed extensively across various specialties for diagnosing disease, assessing anatomical changes, and monitoring treatment efficacy. Beyond these traditional uses, CT also enhances hybrid imaging techniques such as PET/CT and SPECT/CT by providing a basis for patient‐specific attenuation correction and anatomical localization. Given the use of ionizing radiation in CT imaging, protocol optimization is valuable for ensuring diagnostic benefit while minimizing patient risk. Therefore, accurate estimation of patient radiation dose from CT scans is indispensable, as these estimates inform risk assessments and also fulfill regulatory compliance requirement, underscoring their critical role in safe and effective medical imaging.

Estimates of organ absorbed dose delivered by CT imaging can be computed using 3D anatomical models of the patient coupled with radiation transport simulations using experimentally validated source terms for a specific reference CT scanner of a given manufacturer and model, to include the x‐ray energy spectrum, the x‐ray fluence, and its angular distribution across the fan beam. To convert dose estimates from the reference CT scanner to those of a different scanner model and/or manufacturer, the concept of volume computed tomography dose index (CTDI_vol_) scaling can be used as originally proposed by Turner et al.^1^ In the CTDI_vol_ scaling approach, the patient organ dose for the CT scanner of interest $\left(D_{scanner,organ}\right)$ is given as the product of the patient organ dose reported for the reference CT scanner $\left(D_{reference,organ}\right)$ and the corresponding ratio of CTDI_vol_ values for the scanner of interest and that for the reference CT scanner:

(1) $$\begin{array}{c} D_{scanner,organ}=D_{reference,organ}\times \left(\frac{CTDI_{vol,scanner}}{CTDI_{vol,reference}}\right) \end{array}$$

Two currently and widely used computational phantom‐based CT organ dosimetry codes that are based upon the CTDI_vol_ scaling method include NCICT from the National Cancer Institute,^2^ and VirtualDose from Virtual Phantoms, Inc.^3^ The NCICT software utilizes the UF/NCI voxel‐type adult, pediatric, and pregnant female computational phantom series,^4^, ^5^ while VirtualDose is based upon a combination of the RPI adult,^6^, ^7^ and RPI pregnant female voxel phantoms,^8^ and the UF/NCI pediatric voxel phantoms.^4^ NCICT is constructed using collimation‐adjustable axial‐scan organ dose coefficient libraries for its reference CT scanner – the Siemens SOMATON Sensation 16. VirtualDose is based upon this same reference CT scanner for its pediatric phantom series, but uses the GE Lightspeed Pro 16 as the reference scanner for its adult and pregnant female phantoms.

The purpose of the present study is to present a new dataset of normalized CTDI_vol_ (nCTDI_vol_) values for an array of CT scanners currently considered within the MIRDct software, which is now freely available at MIRDsoft.org.^9^ Similar to the NCICT and VirtualDose CT codes, MIRDct is based upon collimation‐adjustable axial‐scan organ dose coefficient libraries for its selected reference CT scanner – the Canon Aquilion One Genesis. The virtual patient models available within Version 1.0 of MIRDct include the full set of twelve ICRP mesh‐type adult and pediatric phantoms in either their arms down or arms up position.^10^, ^11^ The nCTDI_vol_ dataset presented here, as extracted from the user console of each CT scanner, is applied using the methodology of Turner et al.^1^ to scale organ‐dose estimates from this reference scanner to an array of other CT scanners deployed in current clinical use. Comparisons are also made between the nCTDI_vol_ dataset of MIRDct and those currently employed within the NCICT^12^ and VirtualDose^3^ codes for this same subset of CT scanners.

## 2 MATERIALS AND METHODS

### 2.1 Methods of reporting CTDI_vol_

Values of the CTDI_vol_ may be assigned using experimentally derived values of air kerma (mGy) obtained in a 100‐mm pencil ion chamber inserted within a standardized polymethylmethacrylate (PMMA) CTDI quality control phantom of either 16‐cm or 32‐cm diameter (head and body phantom, respectively). For each sized phantom, as well as for each combination of technique parameters such as tube voltage, collimation, and filter size, an air kerma measurement is taken at both a central and at four peripheral positions within the PMMA phantom. Using an average of the peripheral measurements, these two values of CTDI_100_ are then scaled by factors of (1/3) and (2/3), respectively, with the result being the weighted computed tomography dose index, or CTDI_w_. The CTDI_vol_ is the CTDI_w_ scaled inversely by the pitch of the CT scan, as shown in Equation (2):

(2) $$\begin{array}{cc} CTDI_{vol} & =\frac{CTDI_{w}}{pitch}=\left[\frac{1}{3}CTDI_{100,central}\right.\\ & \left.+\;\frac{2}{3}CTDI_{100,periphery}\right]/pitch \end{array}$$

An important aspect of Equation (1) is that the CTDI_vol_ values of the desired CT scanner and the reference CT scanner must both correspond to the same sized PMMA phantom – both at 16‐cm or both at 32‐cm. An alternative estimate is the console‐displayed value of CTDI_vol_, which is pre‐set by the CT manufacturer from in‐factory measurements and is reported for each unique CT image protocol and technique factor combination. An advantage of assembling the CTDI_vol_ library from console‐display settings is consistency across all currently deployed CT scanners of a given make and model.

### 2.2 Collection of CTDI_vol_ data

To assemble the dataset, various scanner models were selected from four major CT manufacturers: Philips Medical Systems, General Electric Medical Systems, Canon Medical Systems, and Siemens Medical Solutions. These manufacturers represent a broad range of commonly used systems in clinical settings. For each scanner, technique parameters were chosen to match closely those used in a previously generated and validated equivalent CT source model of the Canon Aquilion ONE Genesis in PHITS (Particle and Heavy Ion Transport Code System).^13^, ^14^ The parameters selected included the head (16 cm) and body (32 cm) PMMA phantom sizes; bowtie filters, generally grouped into the categories small, medium, and large; tube potentials within the range of 70–150 kVp; and beam collimation settings between 8 and 57.6 mm. All technique parameters used the same effective tube current‐time product of 100 mAs and the same pitch of 1.0 to maintain consistency across scans. For each scanner, the appropriate technique factors and protocols were applied, and the console‐reported CTDI_vol_ values were recorded and normalized to a set value of 100 mAs. The resulting values of this normalization are henceforth referred to as a normalized CTDI_vol_ (nCTDI_vol_). The selected protocols for data collection were chosen primarily to be a generic head or a generic abdomen protocol. Additional protocols were selected when the technique factor combination of interest was not available under either the generic head or abdominal protocols.

### 2.3 Data collection from external databases

For comparison purposes, additional data were gathered from established CTDI databases, including the NCICT and VirtualDose databases. The set of data from NCICT was obtained from their publication regarding CTDI_vol_ information for retrospective dosimetry applications.^2^, ^12^ Data from VirtualDose were collected directly from the software user interface for CT scanners matching those for which data were collected for the MIRDct database.^3^

### 2.4 Analysis of nCTDI_vol_ data

To integrate the data into the MIRDct framework, nCTDI_vol_ ratios were calculated and applied to a pre‐computed Monte Carlo organ dose library, which was based on the ICRP mesh‐based reference adult and pediatric computational phantoms.^10^, ^11^ Of note, not all models from the chosen vendors had technique factors available that exactly matched those available for our reference Aquilion ONE Genesis scanner. For instance, Canon scanners have an upper tube voltage of 135 kVp, while other manufacturers use 140 kVp. For such cases, the closest matching reference technique combination was chosen to compute the appropriate nCTDI_vol_ scaling ratio. Averages and standard deviations of normalized CTDI_vol_ were calculated for each manufacturer across different values of tube voltage.

## 3 RESULTS

### 3.1 Data collection

Normalized CTDI_vol_ values were collected from four manufacturers as noted. Table 1 summarizes the number of scanner models from each of the four manufacturers included in the MIRDct code. The final database of normalized CTDI_vol_ (mGy/100 mAs) values from the 17 scanner models in MIRDct v1.0 for varying combinations of technique parameters, including tube voltage, beam collimation, CTDI phantom size, and bowtie filter size, can be found in the digital Supplementary Annex (under the “MIRDct” tab). All values were normalized to a tube current‐time product of 100 mAs and a pitch of 1.0, and thus nCTDI_vol_ values are numerically equivalent to values of CTDI_w_. Data for nCTDI_vol_ were also collected from both the NCICT and VirtualDose codes. From NCICT, data for 162 scanner models at 10‐mm collimation and four tube voltages were abstracted from published values.^12^ For VirtualDose, data for all 17 scanner models matching those present within the MIRDct database were recorded directly from the VirtualDose software interface. All CTDI_vol_ data collected from both NCICT and VirtualDose are also given in the digital Supplementary Annex (under the respective “NCICT” and “VirtualDose” tabs).

**TABLE 1:** CT scanner models in MIRDct for which CTDI data were collected.

Manufacturer	Scanner model
Canon	Aquilion ONE
	Aquilion ONE Genesis
	Aquilion ONE Prism
	Prime
	Vision
GE	Discovery CT750 HD
	Discovery RT
	LightSpeed 16
	Lightspeed VCT
	Optima 660
	Revolution CT ES
Philips	Brillance iCT
	iCT 256 Slice
Siemens	AS20
	Definition Flash
	Definition Edge
	Somatom Force
Total	17

### 3.2 CTDI across different databases

The normalized CTDI_vol_ values for two CT scanner models, as recorded by three different CT dosimetry codes: MIRDct, NCICT, and VirtualDose, are presented in Table 2. The results demonstrate some variability in the nCTDI_vol_ values across different software platforms, with slight differences observed for identical scanning protocols. The agreement shown is well within the requirement that measured values of CTDI_vol_ for the head and body phantoms shall not deviate from the manufacturer's value by more than ±20%.^15^

**TABLE 2:** Normalized CTDI_vol_ values (mGy/100 mAs) for different scanner models across the three CT Dosimetry codes: VirtualDose, NCICT, and MIRDct for 10 mm collimation and a medium bowtie filter.

Model	Phantom	kVp	MIRDct	NCICT	VirtualDose
Canon Prime	Head	80	8.25	8.21	8.99
		100	15.2	15.3	16.8
		120	23.6	24.1	26.4
		140	30.8	33.5	1^a^
	Body	80	3.40	3.33	3.64
		100	6.55	6.54	7.17
		120	10.5	10.7	11.7
		140	14.1	15.4	15.5
GE Lightspeed 16	Head	80	7.5	7.62	7.42
		100	13.5	13.6	13.6
		120	20.9	20.7	20.9
		140	28.6	28.8	29.2
	Body	80	3.39	3.3	3.12
		100	6.42	6.7	6.80
		120	10.7	10.4	11.6
		140	14.7	15.5	16.0

^a^ The value of 1 displayed in VirtualDose appears to be a placeholder value for missing numbers within the software and appears elsewhere in the reported CTDI values.

### 3.3 CTDI over time

**FIGURE 1:**
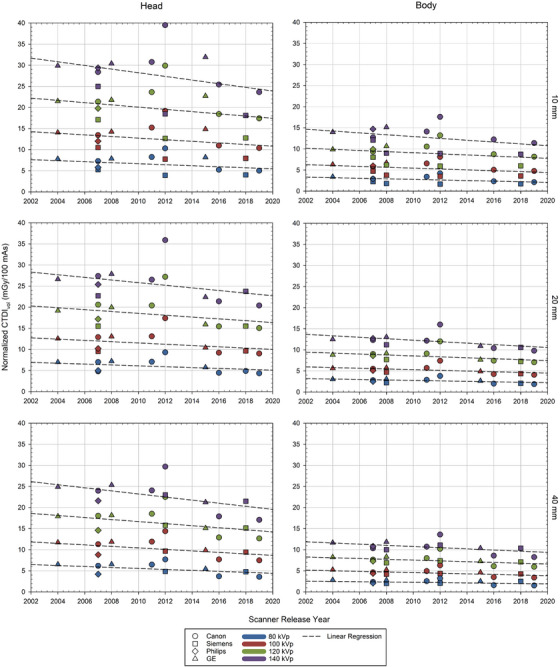
Trends in the 16‐cm head and 32‐cm body phantoms normalized CTDI_vol_ (mGy/100 mAs) versus the release of scanner models included within the MIRDct database.

In Figure 1, nCTDI_vol_ values for the 16‐cm head phantom and 32‐cm body phantom are plotted against the nominal release date for each of the 17 scanner models included within MIRDct. For both phantom sizes, the data exhibit a modest but consistent gradual reduction over time (as newer scanner models were introduced over the period 2002 to 2020). A linear regression was applied to each dataset to illustrate this overall downward trend, rather than implying a constant (horizontal) behavior.

### 3.4 Variation in nCTDI_vol_ across different CT manufacturers

**FIGURE 2:**
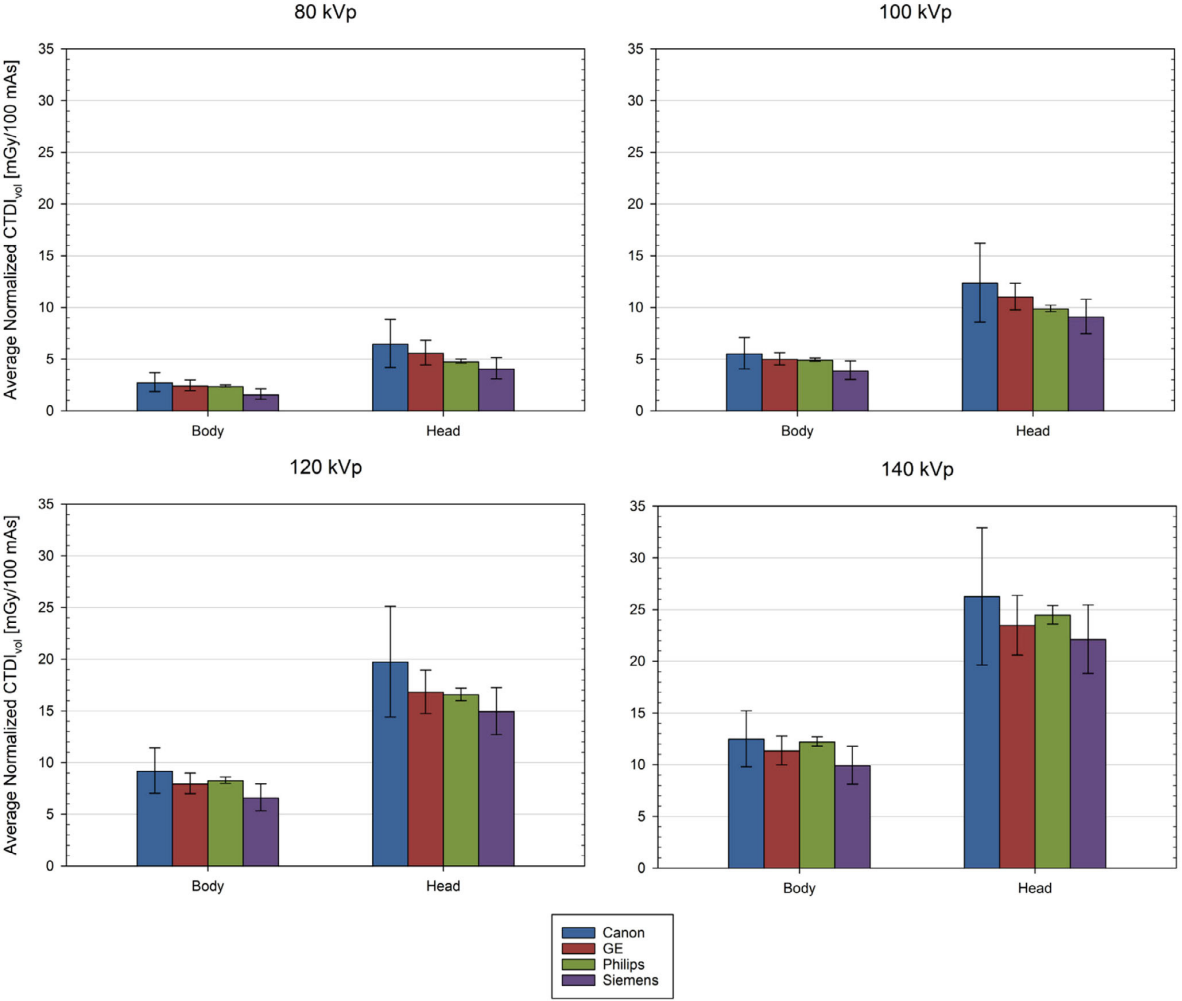
Average values and their standard deviation for normalized CTDI_vol_ for four different kVp values for each of the four major CT manufacturer.

The averages and standard deviations of the normalized CTDI_vol_ across different manufacturers at different tube potentials for both the head and body CTDI phantom were calculated and analyzed, as shown in Figure 2. From these plots, Canon models trend towards higher normalized CTDI_vol_ values for both the head and body phantoms at all tube potentials, but the differences among the different manufacture models are not significantly different.

### 3.5 Difference in ratios computed using different CTDI phantoms

**FIGURE 3:**
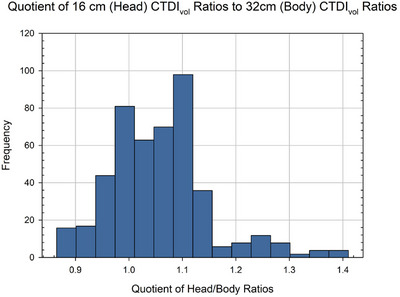
Distribution of the quotients of normalized CTDI_vol_ head ratios over normalized CTDI_vol_ body ratios.

Normalized CTDI_vol_ ratios for the 16‐cm head phantom (scanner model to reference scanner model) were divided by their corresponding ratios for the 32‐cm body phantom to produce a quotient of the two ratios so as to assess the difference between them. The distribution of this ratio quotient is shown in Figure 3. The average ratio quotient was found to be 1.06. In MIRDct, one has the option of organ dose scaling by either ratios of head‐phantom nCTDI_vol_ values or body‐phantom nCTDI_vol_ values. The data of Figure 3 indicates that there would be minimal differences in reported patient organ dose, and thus either nCTDI_vol_ ratio (16‐cm or 32‐cm phantom) could be utilized.

## 4 DISCUSSION

This study explores the degree of variation in tube current‐time product normalized CTDI_vol_ values across different databases assembled for the purposes of creating a transparent library of organ dose scaling ratios that underpin the phantom‐based CT organ dosimetry in MIRDct. Overall, differences were shown to be minor across the three CT dosimetry codes considered in this investigation. For example, nCTDI_vol_ values between our database and that for the NCICT code for the Canon Prime scanner agree within ±2% on average, with variations ranging from −2% to +9%. nCTDI_vol_ values between our library and VirtualDose for the same scanner agree within ±4% on average, with variations ranging from 0% to +12% for the same CTDI phantom size and technique factor combination. Any observed differences can likely be attributed to differences in data collection methodology, as a database assembled from retrospective surveys may contain more variation due to data averaging required in the merger of data from various sources. For consistency across CT scanners currently in clinical practice, our approach was to collect manufacturer pre‐set CTDI_vol_ values from the console display of each CT scanner model in MIRDct.

Normalized CTDI values of all manufactured scanners are shown to have gradually decreased over time, a trend plausibly attributed to advancements in CT technology over the past several decades and in response to initiatives such as Image Wisely to reduce CT patient dose. Innovations such as improved detector quantum efficiency, adaptive scanning protocols, and new reconstruction techniques have reduced radiation dose from CT examinations without compromising diagnostic image quality. Ongoing monitoring of nCTDI_vol_ trends across various clinical contexts continues to be vital for ensuring the continuance of dose optimization across different imaging protocols and patient populations.

The variations observed in this study in nCTDI_vol_ across different CT manufacturers, models, and collected databases, although relatively small, can be attributed to several factors. The implementation of certain scanner design features, such as x‐ray tube design, inherent filtration, and bowtie filter construction, all influence the amount of radiation used, leading to different reported CTDI values. Additionally, database‐specific nCTDI_vol_ values may have discrepancies based on how the values were normalized and reported. Variability can be introduced by data aggregation methods, such as different collection methods and averaging techniques. As a result, similar CT technique settings will yield differing normalized CTDI_vol_ values depending on the manufacturer, model, and intended software use.

As shown in Figure 3, the calculation of the quotient of normalized head‐to‐body CTDI_vol_ ratios was performed to investigate the potential variations in reported patient organ dose by the nCTDI_vol_ scaling method when either head nCTDI_vol_ ratios or body‐phantom nCTDI_vol_ ratios are applied. In the MIRDct software, the user may select either the head or body phantom nCTDI_vol_ ratios when translating our computed reference‐scanner organ doses to organ doses for another CT vendor and model. The quotient of normalized head‐to‐body CTDI_vol_ ratios was found to range from just under 0.9 to just over 1.40. The average quotient was found to be 1.06, with most ratios generally grouped within the range of 1.0 to 1.1, indicating strong similarity between the two phantoms nCTDI_vol_ ratios. Despite this computational similarity, the propriety of using either ratio to scale organ doses depends on the region of anatomical interest and the clinical context. The use of ratios produced by the body phantom, for instance, may be more relevant for estimating organ doses for procedures in adult abdominal and thoracic regions, whereas head ratios would be more appropriately applied for organ dose estimations for CT scans in the head and neck region of adults. Additionally, it is important to note that although most ratios fell within the expected range of 1.0 to 1.1, ratios beyond this range were also observed.

The study has two key limitations. First, the chosen method of collecting console‐displayed values limited the research to include 17 scanner models that were accessible to our group. It does not contain all clinically deployed scanners. However, additional scanners can easily be added to the database in the future. It is also important to note that in this work, we did not perform any image quality evaluation – the intent of the data collection and presentation is to quantitatively assess the magnitude and variability of nCTDI_vol_ values across vendors and models and help elucidate a major source of discrepancies in organ dose estimates when using different CT dosimetry software programs. The nCTDI_vol_ database presented here also does not include historically utilized and presently retired CT scanner makes and models. Organ doses from these systems that are no longer clinically deployed can be very valuable for retrospective studies of patient organ doses, as in CT imaging radiation epidemiology studies.

Secondly, the data collection methods employed throughout this study were limited in accounting for variations in nCTDI_vol_ that may have been observed due to differences in manufacturer bowtie filter configuration and protocol selection. Bowtie filter variation amongst CT manufacturers and models, as well as variations in scanner protocol implementation, can have influence on the resulting normalized CTDI_vol_ values. While we did not catalog each bowtie filter and protocol in detail, the data were intentionally grouped by common metrics (e.g., kVp, collimation, etc.). Future work is needed to investigate the influence of these factors within the developed dataset.

While CTDI_vol_ ratios provide a convenient and widely used method for inter‐scanner dose scaling, their use as the sole basis for organ dose correction introduces important limitations. Specifically, the use of CTDI_vol_ ratios does not fully account for differences in x‐ray beam quality and filtration characteristics across different scanners and acquisition protocols. These factors have the potential to noticeably influence the magnitude of organ doses.

To address these limitations, future work should also explore the inclusion of further measurements to account for differences between scanners with very different beam filtrations. Half‐value layers (HVL), the thickness of material (typically copper or aluminum) necessary to reduce an X‐ray beam's intensity by half, is a useful metric of beam hardness, with a higher HVL being indicative of a harder beam produced by an x‐ray spectrum with a higher average photon energy, which more effectively penetrates tissue with less attenuation. Scanners having different HVLs, even for the same kVp, will deliver different dose distributions within a phantom or a patient. Incorporating HVL data, or alternatively values of peripheral and central CDTI_100_ taken within a standardized CTDI phantom, either directly measured or extracted from medical physics quality control assessments, would allow for improved spectral modeling across different tube potentials and filtration schemes. To assess this variability in dose distribution, the ratios of CTDI_vol_ used for scaling Monte Carlo doses from the chosen MIRDct reference scanner may be updated to include additional effects of HVL in the production of scaling factors.

## 5 CONCLUSIONS

A database of normalized CTDI_vol_ (mGy/100 mAs) was created for 17 models of CT scanners from four manufacturers at different tube potentials, collimation settings, x‐ray bowtie filter sizes, and CTDI physical phantom sizes. These nCTDI_vol_ values serve as the basis for cross‐scanner organ dose scaling within the software code MIRDct, a platform for calculating organ absorbed dose using a pre‐computed Monte Carlo‐based library across the ICRP series of mesh‐type reference computational phantoms. The collected values varied across the surveyed models and vendors for similar technique factors. Variation was also observed between the collected nCTDI_vol_ values and previously assembled databases for the CT organ dosimetry codes NCICT and VirtualDose. These variations are attributed to differences in scanner design features, proprietary algorithms, and data collection methods.

## AUTHOR CONTRIBUTIONS

**Laura E. Dinwiddie**: Conceptualization; methodology; data collection; analysis; original draft preparation. **Jared M. Baggett**: Conceptualization; methodology; data collection and analysis; original draft preparation. **James M. Kofler**: Data collection; review of final draft. **Cameron B. Kofler**: Data collection; review of final draft. **Daniel J. Long**: Data collection; review of final draft. **Robert J. Dawson**: Review and editing of final draft. **Stefan K. Wehmeier**: Review and editing of final draft. **Yitian Wang**: Review and editing of final draft. **Juan C. Ocampo‐Ramos**: Conceptualization; methodology; review of final draft. **Lukas M. Carter**: Review and editing of final draft. **Harry Marquis**: Review and editing of final draft. **Gunjan Kayal**: Conceptualization; methodology; review of final draft. **Adam L. Kesner**: Project administration and funding; methodology; review of final draft. **Wesley E. Bolch**: Project administration and funding; conceptualization; methodology; review of final draft.

## CONFLICT OF INTEREST STATEMENT

The authors declare no conflicts of interest.

## ETHICS STATEMENT

This study did not involve human subject research.

## Supporting information



Supporting Information

## Data Availability

All data from this study are provided in the article and in its Supplemental Annex file.
